# Sublingual Dermoid in an Adult Patient

**DOI:** 10.7759/cureus.63178

**Published:** 2024-06-26

**Authors:** Vidit, Manish Jangra, Arnav Gupta, Bhavinder Kumar Arora

**Affiliations:** 1 Department of General Surgery, Pandit Bhagwat Dayal Sharma Post Graduate Institute of Medical Sciences, Rohtak, IND; 2 Department of Neurology, Pandit Bhagwat Dayal Sharma Post Graduate Institute of Medical Sciences, Rohtak, IND

**Keywords:** benign lesions, surgical excision, floor of mouth, sublingual mass, dermoid cyst

## Abstract

Dermoid cysts in the floor of the mouth are a relatively rare and unusual site of location anomalies presumed to be caused by entrapment of germinal epithelium along the lines of embryonic fusion. It presents as soft, non-painful, and slowly growing swelling along the lines of fusion during the closure of mandibular and hyoid branch arches. These cysts are developmental and histopathologically classified into three types: epidermoid, dermoid, and teratoid. We are reporting a rare case of a 32-year-old female who presented in the outpatient department with complaints of painless swelling over the floor of the mouth for two years, suggesting a benign sublingual mass. This case report underscores the importance of clinical presentation, diagnostic workup, and surgical approach in achieving successful outcomes for sublingual mass.

## Introduction

Dermoid cysts that appear on the floor of the mouth are a rare benign congenital anomaly [1]. During embryonic development, either entrapment of the germinal epithelium, i.e., ectodermal, endodermal, and mesodermal elements, or failure of separation of the ectoderm from the mesoderm during the third to fifth weeks of gestation along the lines of embryonic fusion, can occur anywhere in the body [1,2]. About 80% of dermoid arises from the ovaries, testis, and sacral regions [2]. However, dermoid cysts can occasionally present in abnormal locations, such as the floor of the mouth, implying diagnostic and therapeutic challenges. Sublingual dermoid cysts are intensely rare, documented for 1-1.6% of all of them, and are located beneath the tongue [2,3]. Here, we are reporting a case of an abnormal location of a sublingual dermoid cyst.

## Case presentation

**Figure 1:**
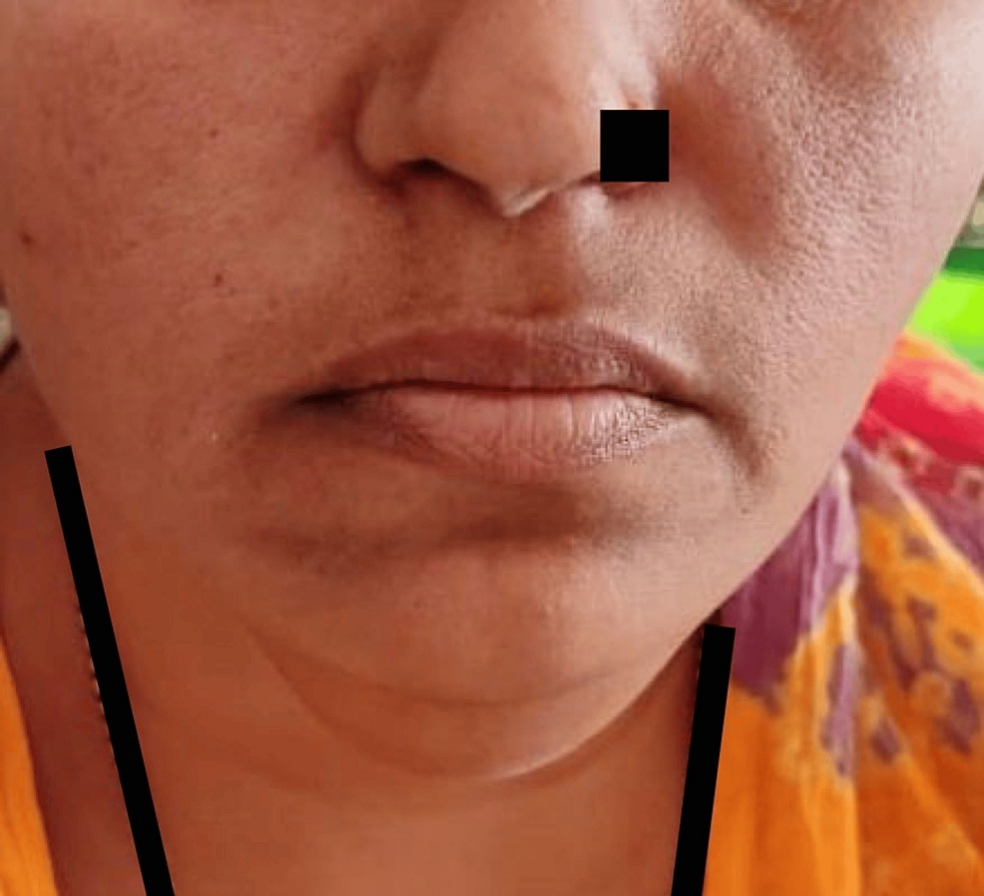
Swelling is seen under the floor of the mouth.

A 32-year-old female presented to the outpatient department with complaints of painless swelling over the floor of the mouth for two years that were sudden in onset, gradually progressive, and intermittent in nature, with discomfort during chewing. She reported no difficulty breathing, speaking, or swallowing. Additionally, there was no history of trauma or previous surgeries in the oral cavity. On local examination, a firm, non-tender, mobile mass was palatable beneath the tongue with no associated erythema or overlying skin changes (Figure 1).

**Figure 2:**
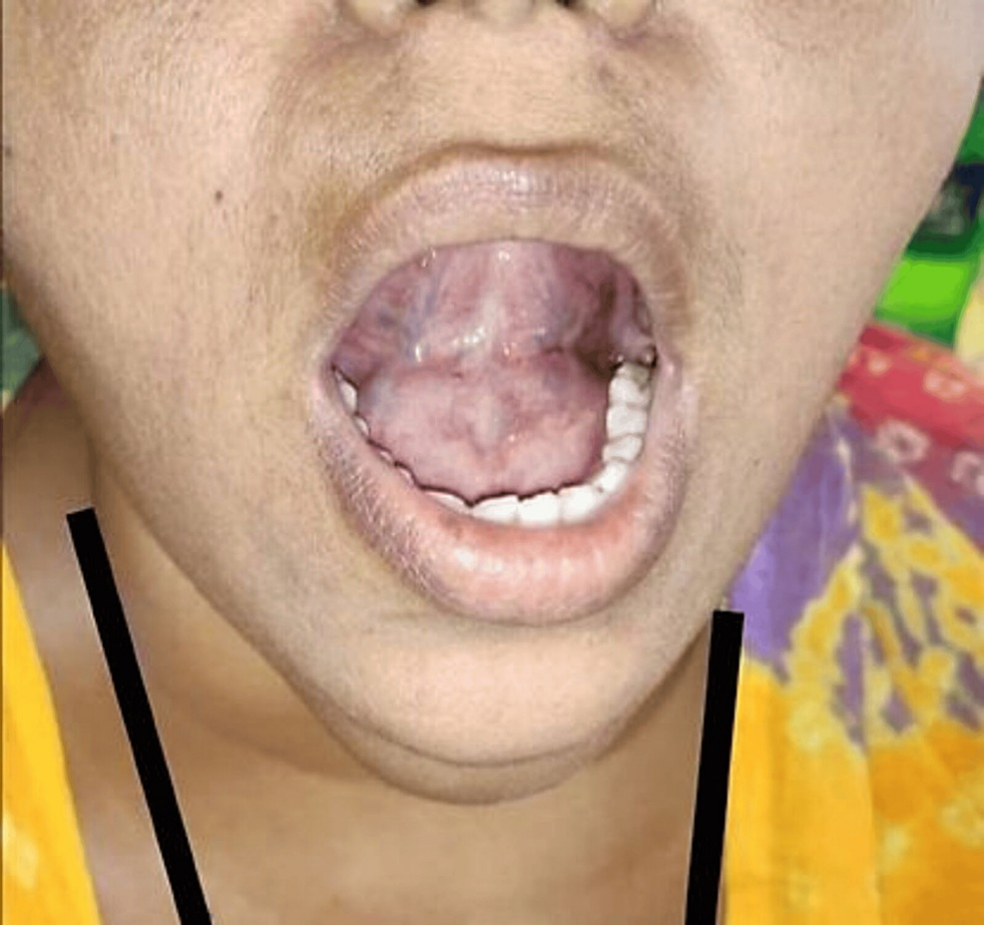
Intra-oral examination shows swelling on the floor of the mouth.

Intra-oral examination revealed approximately 5 cm in diameter submucosal, non-fluctuating mass on the floor of the mouth extending towards submental space with superior displacement of the tongue, normal mucosal integrity, and no evidence of dental abnormalities. A provisional diagnosis of a sublingual salivary gland or dermoid cyst was suspected (Figure 2).

**Figure 3:**
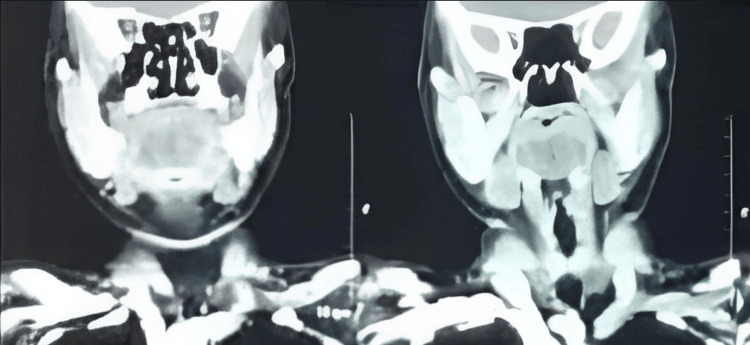
Computed tomography images on the left and right show sublingual swelling.

On further evaluation with radiological imaging modalities, a contrast enhanced computed tomography scan was performed to characterize the lesion and assess its relationship with adjacent structures, suggesting a well-defined, hypodense lesion measuring 5.2cm*2.2cm in the sublingual space, consistent with a cystic lesion (Figure 3).

**Figure 4:**
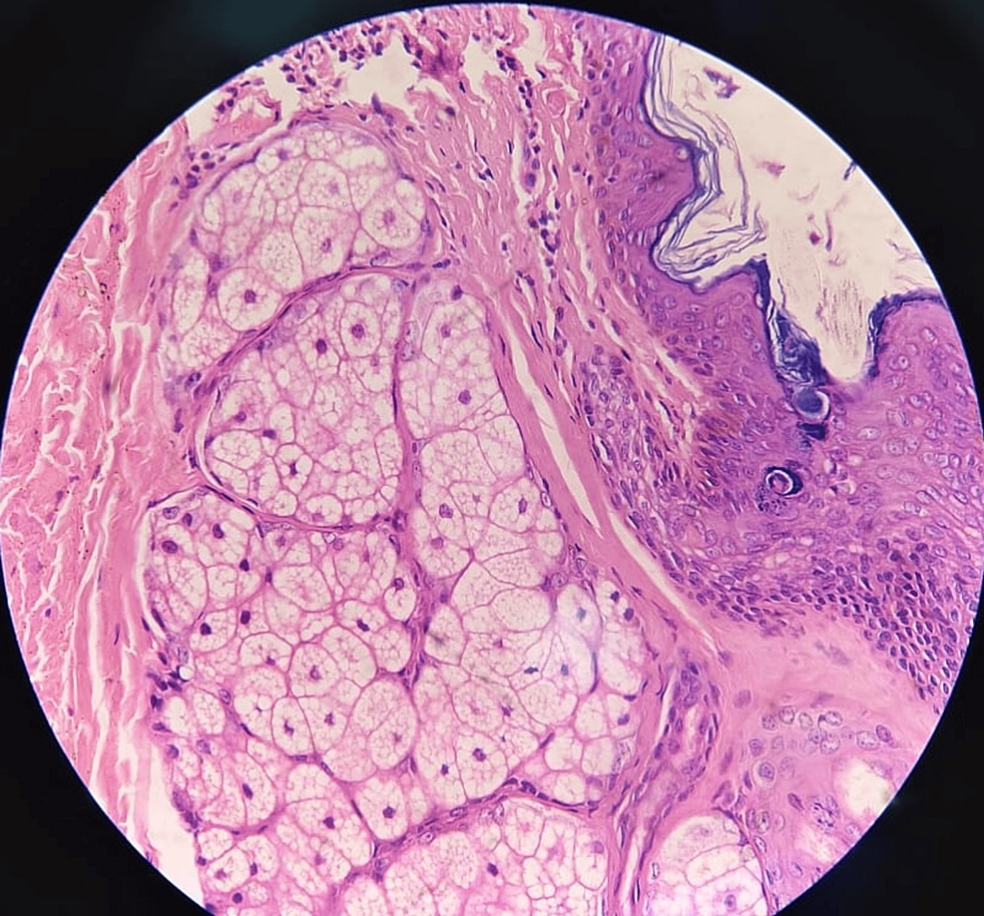
Histopathological image depicting findings consistent with a dermoid cyst.

When keratinous debris was found in the aspirated fluid during fine needle aspiration, cytology suggested the diagnosis of a sublingual dermoid cyst. The patient was counseled and scheduled for a surgical excisional biopsy under general anesthesia through a trans-cervical approach of a sublingual dermoid cyst. In this case, the transcervical approach was chosen by keeping in mind the size of the cyst and accessing the sublingual region to prevent and reduce the risk of injury to vital structures and to provide better exposure. The cyst and its capsule were separated from surrounding tissues by taking care to preserve the lingual nerve and submandibular duct. The intraoperative specimen revealed a cystic structure; the cut section showed sebaceous material and hair follicles, which was consistent with a dermoid cyst. Meticulous hemostasis was achieved, and the surgical site was closed in layers. The patient had tolerated the procedure and had an uneventful postoperative course with resolution of symptoms. She recaptured well and was discharged on the fifth postoperative day with advice for follow-up and medications for one week. Histopathological reporting of intraoperative specimens confirmed the diagnosis of a sublingual dermoid cyst, demonstrating a cavity lined by stratified squamous epithelium with adnexal structures within a cyst wall (Figure 4).

She remained asymptomatic during follow-up visits, with no evidence of recurrence after six months postoperatively.

## Discussion

Dermoid cysts on the floor of the mouth represent relatively rare developmental, though benign, lesions. These cysts originate from ectodermal tissue remnants during embryonic development, are lined with stratified squamous epithelium, and contain a variety of ectodermal derivatives such as hair follicles, sebaceous glands, and sweat glands along the lines of embryonic fusion [1]. They are commonly between the first and second branchial arches during the first four weeks of development [2]. This ectodermal tissue proliferates, forming a cystic structure containing its derivatives anywhere in the body. It is most commonly seen around the ovaries, testis, and sacral regions, but rarely in sublingual regions. Patients are mostly present in infancy, childhood, or the first decade of life. It is occasionally seen in adults with symptoms of difficulty chewing, swallowing, or dysphonia if the cyst grows significantly in size [2,3]. Patients with sublingual dermoid cysts clinically present as painless, slowly and progressively enlarging cystic midline mass beneath the tongue. On palpation, masses are typically smooth, fluctuant, and mobile, with a characteristic doughy consistency suggestive of a cystic lesion. The final diagnosis is made only after a clinical examination supplemented by the radiological and histopathological findings of excision biopsy specimens [3,4]. Differentiation radiological imaging modalities such as ultrasonography, computed tomography, magnetic resonance imaging, etc. supplement confirming the diagnosis and assessing the extent of the cystic lesion with respect to mylohyoid muscle. Ultrasonography is the primary investigation of choice suggestive of a well-defined hypoechoic mass on the floor of the mouth [5]. 

The main goal for the management of a sublingual dermoid cyst is surgical excision. It helps in preventing recurrence and associated complications such as infection, rupture, obstruction of the airway, or swallowing. The approach towards excision depends on the size, location, and extent of the cyst and adjacent structures [6,7]. Smaller ones are managed via a transoral approach, while larger cysts or those involving deeper structures may require a transcervical or combined approach along with adequate cyst margins [7]. The preoperative workup is essential to prevent the risk of injury to adjacent structures such as the lingual nerve, submandibular duct, and major blood vessels [6]. Histopathological examination reports suggest derivatives of the ectodermal, mesodermal, and endodermal epithelium, such as skin appendages, cartilage, teeth, etc. The histopathological examination report of the excised specimen confirmed the nature, type, and content of the lesion derived from different layers and ensured adequate margins during surgical excision.

The prognosis of lesions following surgical excision of sublingual dermoid cysts is excellent, with low recurrence rates [8]. A complete excision of the cystic lesion along with its capsule prevents its recurrence.

## Conclusions

Sublingual dermoid cysts are relatively unusual lesions that clinicians should keep in mind as a differential diagnosis of sublingual masses. Multiple interdisciplinary collaborations for the diagnostic workup, including radiological imaging studies and fine needle aspiration cytology, are essential for accurate diagnosis and treatment planning for favorable outcomes. Surgical excisional biopsy is the gold standard treatment via different approaches depending on the size of the cyst and the involvement of mylohyoid muscle, such as transcervical or transoral. In this case report, a transcervical approach is used for better exposure and excellent outcomes with low recurrence rates. Long-term follow-up is crucial to monitor recurrence and ensure favorable outcomes. This case report highlights the importance of early recognition, preoperative evaluation, and successful management of such unusual lesions to provide timely and effective care to affected individuals. 
